# A temporal assessment of risk of non-indigenous species introduction by ballast water to Canadian coastal waters based on environmental similarity

**DOI:** 10.1007/s10530-023-03019-1

**Published:** 2023-03-04

**Authors:** Ruixin Song, Yashar Tavakoli, Sarah A. Bailey, Amilcar Soares

**Affiliations:** 1 Department of Computer Science, Memorial University of Newfoundland, St. John’s, NF Canada; 2 Great Lakes Laboratory for Fisheries and Aquatic Sciences, Fisheries and Oceans Canada, Burlington, ON Canada

**Keywords:** Ballast water, Environmental distance, Environmental similarity, Non-indigenous species, Risk assessment, Seasonal change

## Abstract

The environmental similarity scores between source and recipient locations are essential in ballast water risk assessment (BWRA) models used to estimate the potential for non-indigenous species (NIS) introduction, survival, and establishment, and to guide management strategies aiming to minimize biodiversity loss and economic impacts. Previous BWRA models incorporate annual-scale environmental data, which may overlook seasonal variability. In this study, temporal variation in sea surface temperature and salinity data were examined at global ports, and the influence of this variation on environmental distance calculations (and corresponding risk of NIS) was examined for ballast water discharges in Canada by comparing outputs from monthly and annual scale assessments in a BWRA model. Except for some outliers in the Pacific region, the environmental distances based on monthly scale data generally become smaller in all regions, demonstrating that the model using annual decadal average environmental data to inform environmental matching can underestimate risk of NIS survival and establishment in comparison to monthly data. The results of this study suggest future evaluations incorporating the date of ballast water uptake and discharge can provide a more sensitive assessment of risk reflecting seasonal variability compared to an annual average risk model.

## Introduction

The biological invasion process can be divided into several stages, including transport, introduction, establishment, and spread (Blackburn et al. 2011), all of which must be successfully passed for a non-indigenous species (NIS) to be considered ’invasive’, which refers to species introduced into new habitats with significant detrimental impacts on native organisms (Davis and Thompson 2000). Overcoming the barriers associated with each stage depends on multiple factors involving propagule pressure (Colautti et al. 2006; Briski et al. 2018), environmental similarity (Barry et al. 2008) and species’ traits (Pyšek and Richardson 2010). The multiple stages and interacting factors are challenging for risk assessment and proactive management, especially in aquatic ecosystems where physical access is limited and data/information are incomplete, unvalidated or not standardized across regions (Ojaveer et al. 2014, 2015). Increased human activities across biogeographic regions have brought the issue of biological invasions to the forefront, with the main vectors for aquatic NIS introduction and spread being ballast water discharge and biofouling on ships (Keller et al. 2011b; Bailey et al. 2020b).

Ballast water has been responsible for the transport and introduction of a variety of aquatic species across many regions, including bacteria, fungi, plants, and animals (Ruiz et al. 1997; Elçiçek et al. 2013; Bailey et al. 2020b). Therefore, a series of ballast water risk assessment (BWRA) tools were launched during the last few decades to guide management activities based on three main approaches: environmental matching, species’ biogeography and species-specific (David et al. 2015). The species-based methods call for a multitude of data such as species’ geographic distribution, life cycle attributes, and physiological tolerances to assess the potential for introduction and establishment in a new environment (Barry et al. 2008). A key issue with species-based approaches is that, by definition, species fundamental niches differ from their realized ones, so there is a need to continually update data based on emerging information with each new species location record. In contrast, the environmental matching strategy is a more general approach, with more readily-available data that do not need such frequent updating. However, given climate change and cyclical climate variability, environmental data should be updated periodically to maintain the validity of the analysis. Furthermore, the environmental matching approach enables the customization of the environmental variables according to the needs of the assessment.

Early BWRA models using the environmental matching approach include a risk assessment in Nordic coastal waters based on salinity and climate factors (Gollasch and Leppäkoski 1999), and the GloBallast BWRA (Clarke et al. 2003; Awad et al. 2004) led by the International Maritime Organization (IMO) which contains more than 30 environmental parameters. More recent examples include an assessment of salinity match between donor and recipient ports for ships traveling between canals and oceans (David et al. 2013) and probabilistic models integrating the environmental matching method to assess the risk of NIS invasion through ballast water (Seebens et al. 2013, 2016; Saebi et al. 2020). Temperature and salinity are consistently included in these models as environmental matching variables, since they are considered the most critical factors contributing to the survival and establishment of aquatic species (Barry et al. 2008; Keller et al. 2011a).

A recent study comparing multiple sets of environmental variables against species distribution data shows that models using fewer, but more relevant, variables can perform better than those including many variables in the environmental matching approach (Bradie et al. 2015). As the most important factors for BWRA, sea surface temperature and salinity can be expressed with different measurements (e.g., minimum or maximum annual temperature (Hoek 1982), annual average salinity, etc.). Since the publication of an influential model using annual average environmental data (Keller et al. 2011a), many subsequent studies have conducted environmental matching assessments following the same variable set, including the BWRA tool currently used by Transport Canada (Bradie and Bailey 2021; Etemad et al. 2022) which is the baseline model in this study. Although prior models are stable and provide insight on the likelihood of NIS introduction, the use of annual averages of temperature and salinity has limitations primarily related to insensitivity to seasonal variability, which potentially affects the probability of any introduced NIS survival and establishment at a given time point.

Therefore, this study uses monthly temperature and salinity values obtained from the World Ocean Atlas 2018 (Zweng et al. 2019; Locarnini et al. 2019) within the baseline model to explore the impact of temporal variation in environmental factors on BWRA, based on a case study of ships destined for Canadian ports in 2019 and 2020. The outcomes of the monthly and annual temporal scales are compared statistically with the null hypothesis that there is no difference in risk estimates using monthly vs. annual values in the calculation of environmental distance between ports. Due to wider seasonal variation in sea surface temperature in temperate climate zones, we predicted that ballast water from temperate ports of origin will show greater variability in environmental distance calculations for monthly vs. annual scale assessments. In addition, the opposite seasons in the northern and southern hemispheres may result in Canadian ports with low overall temperatures being at higher risk of NIS survival and establishment from ballast water originating from the southern hemisphere winter. To explore these hypotheses, we calculated risk values for each pair of ports during different months of the year using fixed time intervals and explored the interannual risk variability.

## Materials and methods

### Fundamentals of the baseline risk model

The baseline model used in this study (Bradie and Bailey 2021) is the practical tool used by Transport Canada for assessing ballast water risk as an essential input into decisions concerning derogation requests and contingency measures (Etemad et al. 2022). Canada requires ships to submit ballast water reporting forms, declaring the source port of any ballast water to be discharged in Canadian waters, as well as details about any management activities undertaken (e.g. ballast water exchange and/or ballast water treatment). The baseline BWRA model assesses the risk of each ballast water tank discharge by comparing environmental similarities between source and recipient ports (Bradie and Bailey 2021). The model first normalizes the environmental data with a z-score procedure applied to four environmental variables: (i) maximum, (ii) minimum, (iii) average temperature and (iv) average salinity.

More formally, the environmental vectors *V* are:

1 $$\begin{aligned} V = \langle T_{max}, T_{min}, T_{avg}, S \rangle \end{aligned}$$

where $T_{max}, T_{min}, T_{avg}$ are the normalized maximum, minimum and average temperature, and *S* is the normalized average salinity of a source or destination location. After, the Euclidean distance is calculated between the four variables for ballast water source ($v_s$) and destination ($v_d$) ($v_s$, $v_d$ $\subset V$) as follows:

2 $$\begin{aligned} env\_distance(v_{s_i},v_{d_i}) = \sqrt{\sum _{i=1}^{|V|}(v_{s_i} - v_{d_i})^2} \end{aligned}$$

Ballast water management actions that could alter the environmental variables, such as offshore ballast water exchange, are not considered in the model since the tool is used as part of a precautionary management approach considering the ’worst-case’ scenario. The assessment can easily be re-executed using geographical coordinates of ballast water exchange as the source location when desired.

As previously described, the baseline model currently uses annual-scale environmental data—mean temperature during the warmest month (as the maximum temperature), mean temperature during the coldest month (as the minimum temperature), annual average temperature and annual average salinity, following Keller et al. (2011a). Risk categories are then assigned based on the distribution of environmental distances between all pairwise permutations of ports on a global scale. The distribution of distance values is categorized by the percentiles in Table 1.

**Table 1:** Percentiles of environmental distance values and corresponding risk categories based on all possible combinations of global port pairs

Percentile	Distance value *d*	Category
0–20%	$d<0.787$	Very high risk
20–40%	$0.787 \le d < 1.500$	High risk
40–60%	$1.500 \le d < 2.778$	Moderate risk
60–80%	$2.778 \le d < 4.020$	Low risk
80–100%	$d \ge 4.020$	Very low risk

Transport Canada personnel can use these categories as part of prioritization to quickly identify ballast tanks that pose greater risk since categorical data are more easily interpreted than the numerical distance values. In this study, however, only numeric distance values are used in the analysis because the data are continuously distributed and have a wider range of values than the categorical results.

### Compilation of monthly and annual environmental data

The compilation of the monthly-scale environmental data was conducted using two datasets: (i) a list of 8392 global shipping ports with positional coordinates (latitude and longitude) (Bailey et al. 2020a) and (ii) monthly sea surface temperature and salinity data downloaded from the World Ocean Atlas 2018 (WOA 2018), available from the National Centers for Environmental Information (NCEI) as the average of six decadal means from 1955 to 2017 following systematic data quality control techniques (Locarnini et al. 2019; Zweng et al. 2019). The environmental variable values were available at a one-degree grid resolution (i.e., points spaced at approximately 111 km) from January to December.

The shipping port locations were matched with sea surface environmental variables based on closest geodesic distance. As the distance for some inland ports to the nearest environmental data point was greater than 2 grid cells (greater than 222 km), the analysis was restricted to coastal ports best represented by the data (all ports farther than 2 grid cells from the nearest environmental data point were excluded, e.g., Laurentian Great Lakes’ ports). The match procedure constructed 24 intermediate layers covering 12 months’ salinity and temperature data. In each layer, sea surface values were missing for 0.5–3% of the 31,000–33,000 environmental data points. Since the percentage of missing values was relatively small, these points were dropped for each layer and the closest match procedure was rerun. The layers were then combined to create a dataset of global shipping ports with monthly environmental values.

The standard deviation (STDEV) of the 12 months’ environmental values was calculated at each port to examine how the environmental variables change during the year on a monthly basis. The STDEVs at the ports were used to generate a raster layer, and the equal interval method was applied to categorize the values into equal bins.

### Evaluation of monthly versus annual environmental distances for ballast water discharges in Canada

First, we extracted ballast water records (i.e., location and dates when ballast water was taken up and discharged, for individual ballast water tanks) from ballast water reporting forms submitted by ships entering Canadian waters in 2019 and 2020, as stored in the Canadian Ballast Water Information System (Etemad et al. 2022). The tank records for the two years were processed separately to see if there was a similar/stable pattern across years. Next, we calculated environmental distance values for each pair of ballast water source-recipient locations using annual and monthly environmental data as inputs to the baseline model and created density distribution plots to visualize the difference between the two temporal scales in each year.

We then calculated the difference in environmental distance values produced using the monthly and annual environmental datasets, subtracting the annual distance from the monthly distance for each tank record: $distance\_diff = env\_distance_{month} - env\_distance_{year}$. The resulting difference values were divided into two sets, one with positive difference values and the other with negative difference values. Positive difference values result when a port-pair was at lower risk (had a greater environmental distance) using the monthly environmental data, while negative difference values result when the port-pair was at higher risk (had a lower environmental distance) with the monthly environmental data. These two sets of difference values were examined separately, selecting the 75% and 90% percentiles of positive and negative differences as thresholds of importance, generating four categories:

1. (Positive difference greater than 1.586)—port-pairs with much lower risk using monthly environmental data
2. (Positive difference between 0.835 and 1.586)—port-pairs with lower risk using monthly environmental data
3. (Negative difference between $-$1.720 and $-$2.248)—port-pairs with higher risk using monthly environmental data
4. (Negative difference lower than $-$2.248)—port-pairs with much higher risk using monthly environmental data

Difference values were averaged across all individual ballast tanks discharged at each Canadian recipient port, and those falling within the above categories were marked in darker colors on a map to visualize ports with more pronounced differences in environmental matching at the two temporal scales. Ports with average difference values outside these categories were marked with lighter colors on the map, indicating ports without notable changes in assessed risk after using monthly environmental data.

In addition to the categorical assessment of the pronounced differences, statistical tests were conducted to evaluate the significance of the overall difference between the monthly and annual environmental distances. Since the distribution plots showed that the distributions were skewed, non-parametric tests were used. Monthly and annual environmental distances were calculated for each port pair in the ballast water tank data, pairing the monthly distance value (based on actual date of the ship trip) with the annual distance value one to one (i.e., the baseline model was run for each ballast tank source-destination record using both scales of environmental data). The Wilcoxon signed-rank test for paired samples (Wilcoxon 1992) was used to examine whether the differences in the two calculations were statistically significant, with the null hypothesis that the differences between the two samples were symmetric about a real number $\mu$ such that the two samples can be recognized as similar distributions. We used the function “wilcox.test” in R (R Core Team 2021) to perform the evaluation with a significance level $\alpha$ of 0.05. The Wilcoxon test effect size, function “wilcox_effsize” in the *rstatix* package (Kassambara 2021), was used to examine the strength of the differences across all paired samples together and for paired samples aggregated by region (Atlantic, Pacific and Arctic). The statistical tests were performed on the paired environmental distances in 2019 and 2020 separately to verify whether the patterns of differences in environmental distances were stable across these two years.

### Standardized analysis of monthly environmental distance variation

Since the statistical analysis conducted in Sect. 2.3 may be biased by specific factors in the Canadian ballast water data such as shipping intensity between specific port pairs or the actual date (month) of different ship trips, a standardized analysis was conducted which excluded replicate tanks and examined differences in monthly vs. annual environmental distances across each unique source and destination port pair across all months in the year (rather than only for the dates of actual ship trips in the Canadian dataset). We calculated the average voyage time ($\tau$) for each unique port pair based on dates reported in the ballast water data.

We then cycled the start date of the voyage from January to December, using $\tau$ as a fixed time interval to calculate 12 environmental distance values representing a one-year cycle for each port pair using Eq. 2 with the corresponding monthly environmental data. The standard deviation of the 12 environmental distance values was then calculated for each port pair to explore the magnitude of change in environmental distance during one year. Furthermore, the source ports were grouped into regions to explore patterns in environmental distance differences by region across months. The country code and regions used followed the ISO-3166 Standard (ISO 2020).

## Results

### Temporal changes in environmental variables at global ports

**Fig. 1:**
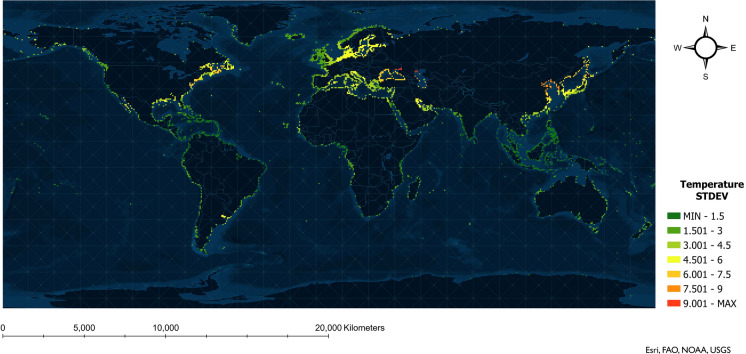
Temporal change in temperature at global coastal shipping ports, illustrated by standard deviation (STDEV) of monthly decadal average values. STDEVs close to zero (dark green) indicate less change in temperature during a year, while large STDEVs (red) indicate greater temperature change

**Fig. 2:**
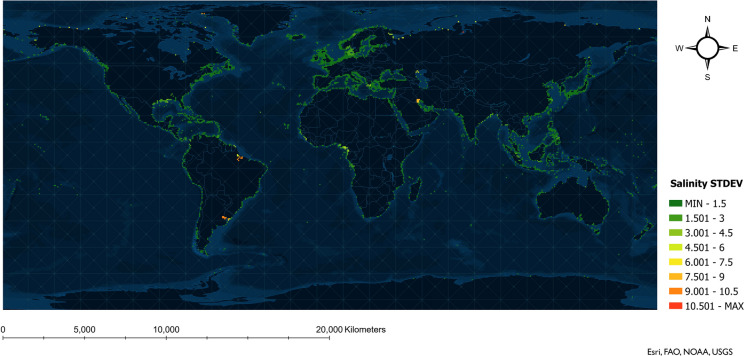
Temporal change in salinity at global coastal shipping ports illustrated by standard deviation (STDEV) of monthly decadal average values. STDEVs close to zero (dark green) indicate less salinity change during a year, while large STDEVs (red) denote greater salinity change

The standard deviation of monthly decadal average environmental values at global coastal shipping ports across one year can be seen in Figs. 1 and  2, for temperature and salinity, respectively. Figure 1 shows that temperature changes greater than three standard deviations occur broadly and are greatest in the northern hemisphere, especially in the temperate climate zone. Figure 2 shows that the largest temporal changes in salinity are mainly concentrated in the estuaries of large rivers (e.g., Amazon and Uruguay rivers in South America, Volga River in Eastern Europe).

### Temporal changes in environmental distance across ballast water discharges in Canada

**Fig. 3:**
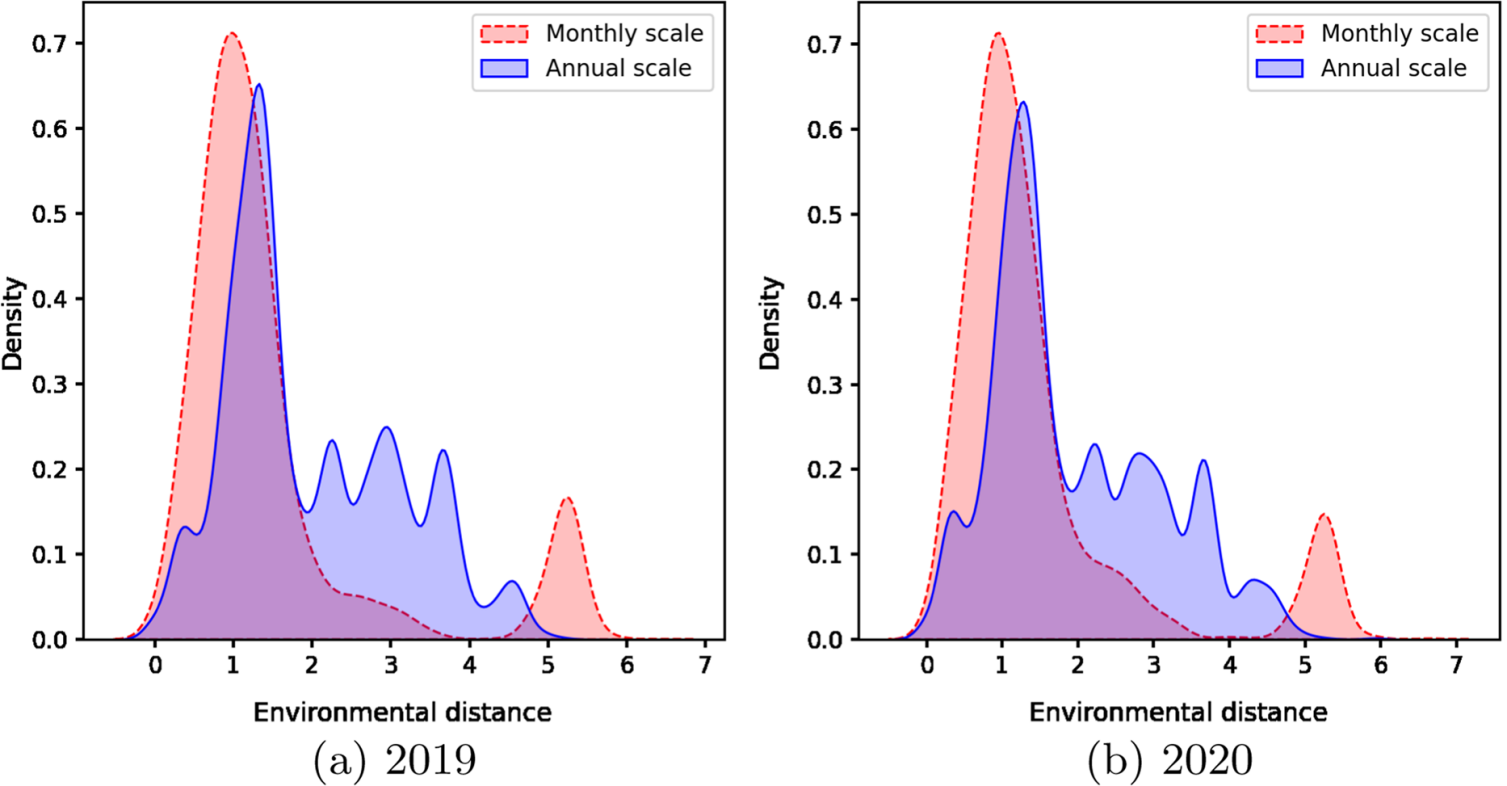
Density distribution plots of environmental distance values calculated using monthly and annual decadal averages for ballast water discharges in Canada in (**a)** 2019 and (**b)** 2020

Ballast water source and discharge locations were extracted from 87,951 tank records (7242 trips) submitted by ships arriving in Canadian waters in 2019 and 2020 (Etemad et al. 2021). After removing inland ports and discharge locations outside of Canadian water, 51,945 tank records (representing 6308 ship trips and 1357 unique source-recipient port pairs) remained for analysis. Figure 3 shows the distribution of the environmental distance values produced by the baseline model using annual and monthly environmental data for all ballast tanks discharged in Canadian waters. The distributions of environmental distances in both years show more extreme values when using monthly data (i.e., monthly distributions have more small and large values).

Looking only at the extreme values in the 90% percentile categories, 17,291 tank records are at very high risk, of which 50.03% and 49.96% are destined for the Atlantic and the Pacific regions, respectively. Meanwhile, 71.50% of 7490 very low-risk tank records were discharged in the Pacific region. Comparing the output of the baseline model using monthly vs. annual environmental data, the proportion of very high risk to very low risk tank discharges increases to nearly 7:3 (monthly) compared to 5:7 (annual).

**Fig. 4:**
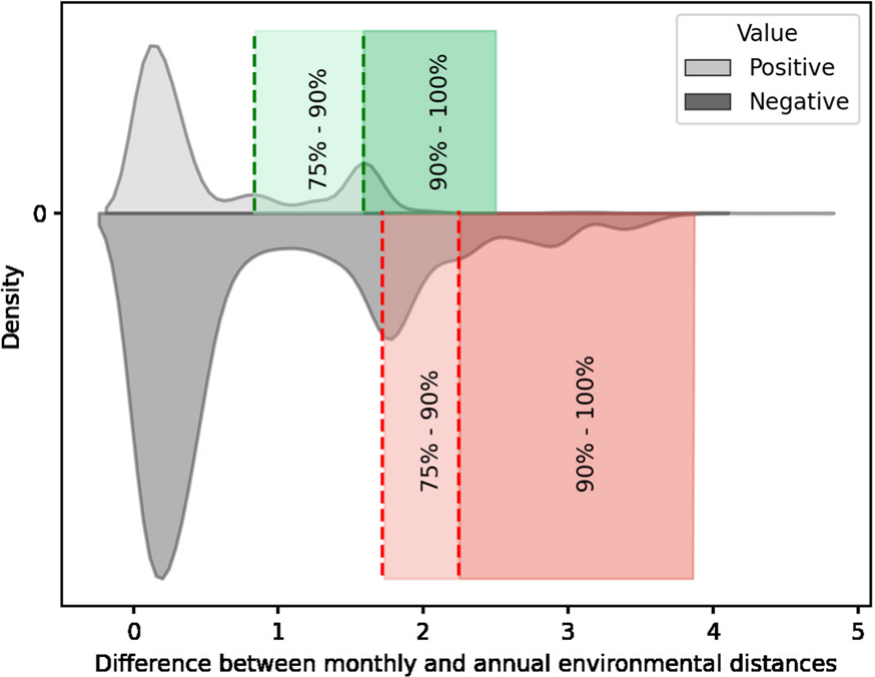
Density distribution plot of the differences in environmental distance calculated using monthly and annual decadal average environmental data, with 75% and 90% percentile categories considered as being a significant change marked (dotted lines). Positive values above the zero-axis represent lower risk using monthly environmental data, while negative values below the zero-axis represent higher risk compared to estimates using annual environmental data

Figure 4 shows differences in monthly and annual average environmental distances. Positive difference values are records where environmental distances increase (i.e., risk values decrease) after using monthly environmental data. Correspondingly, negative difference values indicate records where the environmental distances decrease (i.e., risk values increase).

**Fig. 5:**
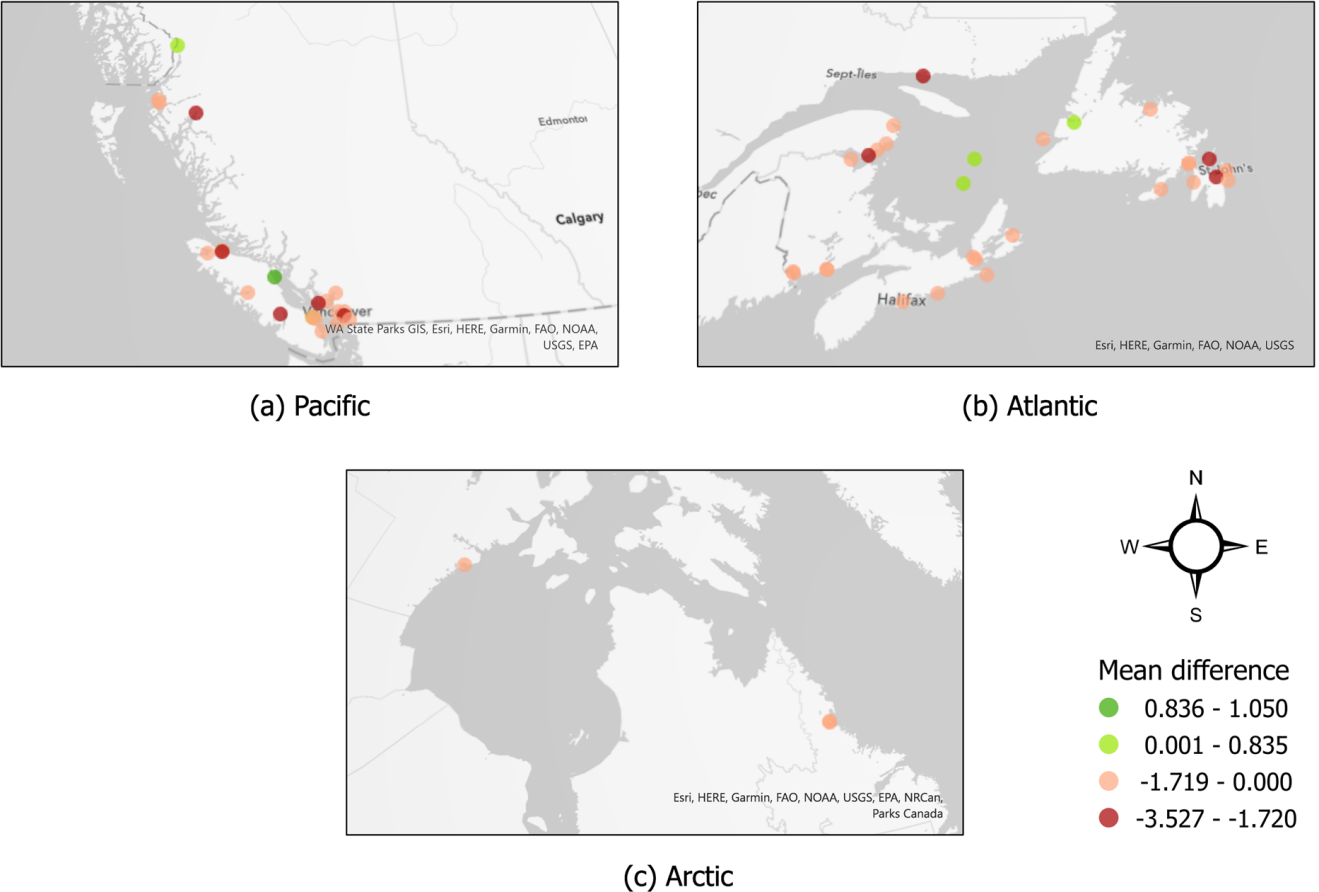
The average differences between monthly and annual scale environmental distance values at Canadian destination ports. The difference values are attributed to four categories according to the percentile 75% significance thresholds as shown in Fig. 4 for the colored areas. Ports marked with orange and dark red have higher risks, while those with light and dark green have lower risks using monthly-scale model

Examining these differences spatially, we observed that the cumulative risk across all tank discharges at individual ports can become much higher using monthly environmental data (e.g., Fig. 5, in dark red: Kitimat, Port McNeil, Port Alberni, Sechelt, New Westminster on the Pacific coast and Havre St. Pierre, Paspébiac, South Brook, Holyrood on the Atlantic coast). Conversely, the cumulative risk becomes much lower using monthly environmental data for only one individual port (Fig. 5, in dark green: Campbell River on the Pacific coast).

Table 2 shows the degree of differences between the paired estimates of environmental distance based on monthly and annual data across regions, using the Wilcoxon signed-rank test with effect size *r*, where the objective of this test is to validate whether there are significant differences between the assessed monthly and annual average environmental distances. The effect size *r* used to measure the size of difference is largest for the Arctic region, followed by the Atlantic region, while being relatively small for the Pacific region. As the *r* values for 2019 and 2020 are very similar, the regional patterns in the risk differences are stable across the two years.

**Table 2:** Wilcoxon signed-rank test results comparing environmental distance values based on monthly vs. annual environmental data for ballast tank discharges in Canada during 2019 and 2020

Region	2019			2020		
	*N*	*r*	Magnitude	*N*	*r*	Magnitude
Pacific	16,574	0.282	Small	18,027	0.267	Small
Atlantic	8294	0.637	Large	8989	0.640	Large
Arctic	45	0.863	Large	16	0.845	Large

*N* is the sample size (# of tank records); *r* is the effect size that quantitatively measures the difference between the paired values, ranging from 0 to 1 where large effect size suggests significant difference. *magnitude* categorizes the effect size as: $<0.3=$ “small”, $0.3 - 0.5=$ “moderate”, $>0.6=$ “large”

**Fig. 6:**
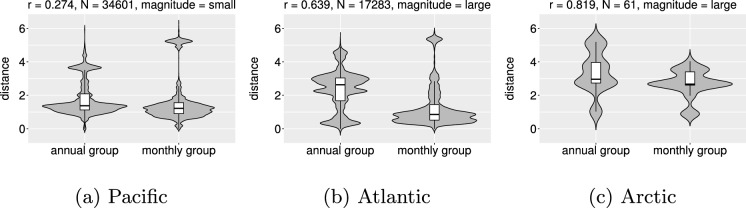
Violin plots showing the distribution of annual and monthly scale environmental distances, by region (panels a–c = Pacific, Atlantic, Arctic, respectively). The vertical black lines show the 1.5 times interquartile range, with white boxes showing the median (center black horizontal line), first and third quartiles (lower and upper box edges, respectively). *r* is the effect size, *N* is sample size of the paired distance values, *magnitude* is based on *r* value

Figure 6 shows the regional distribution of environmental distance values calculated in the baseline model using annual vs. monthly environmental data for the two years of study. Except for some outliers in the Pacific region, the environmental distances based on monthly data generally become smaller in all regions, revealing that when using the monthly data, there is a higher estimated risk of NIS survival and establishment.

### Temporal variation across unique port pairs

**Fig. 7:**
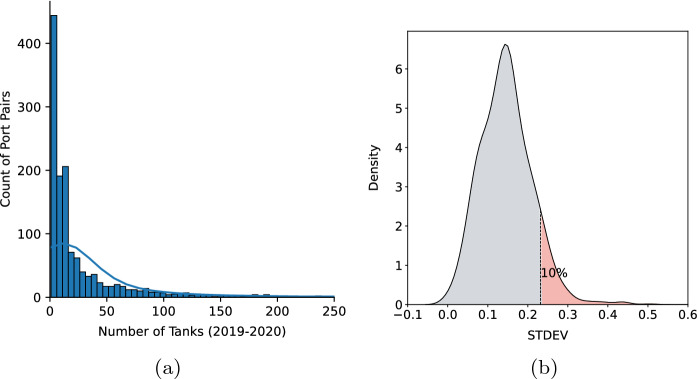
**(a)** Distribution of the number of ballast water tanks (x-axis) connecting individual port pairs (y-axis). Port pairs with more than 250 tank connections were excluded from the plot (about 2.6%) for visualization purposes (listed in Table 4). (**b)** Distribution of standard deviation of environmental distances for all unique port pairs during the 12-month standardized analysis. The red area denotes the 10% of port pairs with the largest STDEVs

The Canadian ballast water dataset contained 1357 unique port pairs after the removal of duplicate trips/tanks with the same source-discharge combinations. Most port pairs are connected by only a small number of ballast tank discharges, while a small number of port pairs have a large number of connections (Fig. 7a). Port pairs with more than 250 tank connections during the two years are listed in Table 4 in Appendix 1. The STDEVs of environmental distance values calculated for all unique port pairs across the 12 months of the year are shown in Fig. 7b, where large STDEV equates to higher variation in environmental distances during a year. Port pairs having both a large number of ballast tank connections (more than 250 discharges) and high variation in environmental distance during the 12 months of the year (top 10% as shown in the red area of Fig. 7b) are presented in Table 3. All of these high intensity/high variability port pairs link Eastern Asia to ports located in the Pacific region of Canada.

**Table 3:** Port pairs with high number of ballast tank connections in 2019–2020 and high variation in environmental distance values using monthly scale

Source region	Source port	Recipient port	Number of tanks	STDEVs
Eastern Asia	Zhoushan	Vancouver (CAN)	854	0.235347
Eastern Asia	Rizhao	Vancouver (CAN)	615	0.255766
Eastern Asia	Qingdao	Vancouver (CAN)	608	0.251918
Eastern Asia	Shanghai	Vancouver (CAN)	395	0.253708
Eastern Asia	Lianyungang	Vancouver (CAN)	370	0.255404
Eastern Asia	Lanshan	Vancouver (CAN)	363	0.268970
Eastern Asia	Dangjin	Roberts Bank	275	0.237261
Eastern Asia	Caofeidian	Vancouver (CAN)	259	0.242195

**Fig. 8:**
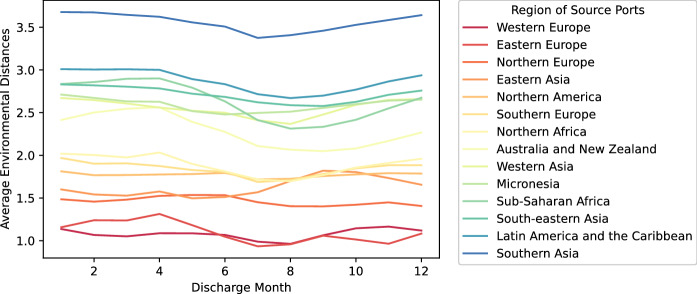
Average environmental distances across unique port-pair combinations during the 12 months of the year, grouped by source port region

**Fig. 9:**
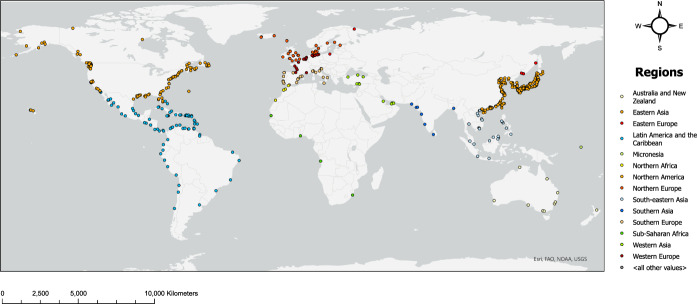
Regional distribution of 602 source ports categorized by 14 regions

Further exploration of monthly environmental distance variation by source port region shows how environmental distance can change during the year (Fig. 8). The spatial distribution of the 602 source ports across 14 global regions is shown in Fig. 9 in Appendix 2.

Overall, for the Canadian destination ports included in this study, it is clear that the lowest environmental distances (highest risk for NIS survival and establishment) are associated with source ports at similar latitudes in Europe, Eastern Asia and North America (Fig. 8).

## Discussion

This study examined the temporal variation of temperature and salinity at ports worldwide and quantified the influence of this variation on environmental distance calculations as well as the corresponding risk for the introduction and establishment of aquatic NIS in Canadian waters. To do so, the use of monthly vs. annual average environmental data was considered within a baseline BWRA model. Except for some outliers in the Pacific region, the environmental distances based on monthly scale data generally become smaller in all regions (Fig. 6), demonstrating that the model using annual decadal average environmental data to inform environmental matching can underestimate risk of NIS survival and establishment in comparison to monthly data, at least for the combination of source-recipient port pairs occurring across Canada. Moreover, the distribution of the monthly vs. annual average environmental distances (Fig. 3) and the statistical comparison results (Table 2) follow the same pattern for both years, suggesting that the results are stable and can be generalized through time.

Spatial examination of differences between the monthly and annual average environmental distances shows that the cumulative risk across all tank discharges at individual ports can become much higher using monthly environmental data in the baseline BWRA model, with only a few ports experiencing a decrease in risk. Further, the assessment of monthly environmental distances for unique port pairs at fixed intervals throughout the year allows for an analysis of year-round risk variability for each port pair. Combined with the sources of ballast tanks, this study further explores the link between environmental conditions in the ballast water source regions and NIS survival and establishment risk.

Although the overall risk increases at most Canadian ports when using monthly environmental data, the regional statistics comparing monthly and annual average environmental distances show an uneven distribution of ballast tank discharges with higher and lower risk values. Some individual ports with increased and decreased risk are adjacent to each other because of the receipt of ballast water sourced from a specific location. For example, the only port with a markedly reduced risk, Campbell River, received only a small number of tank discharges from two U.S. ports. In addition, the proportion of high-risk tanks discharged in a region can affect the result of statistical comparison (i.e., Wilcoxon effect size) for that region. For example, the ballast tank discharges in the Pacific contributed nearly 50% of all ‘very high risk’ extremes but represent only a small proportion of the total ballast tank discharges in the Pacific region, resulting in a small effect size in this region (Table 2).

In correspondence with a previous study which considered risk variation in discharge ecoregions (Seebens et al. 2013), this study incorporates regional information for the source ports, enabling analysis of monthly risk variation in ballast water from specific sources and identification of additional risk patterns. The results indicate that for Canadian recipient ports, the overall invasion risk is higher when ballast water comes from ports at similar latitudes (e.g., Northern and Western Europe) and lower when coming from the tropical zone (e.g., Southern Asia and Latin America and the Caribbean) (Fig. 8). Combined with the temporal variation of environmental variables (Fig. 1), it can be also observed that the risk variation between port pairs often corresponds to larger interannual temperature variations—such as observed along the Mediterranean coast and northeast Asia. This finding is consistent with our hypothesis that ballast water from the temperate zone may have greater variability in assessed risk due to the large interannual variability in sea surface temperature in the temperate climate zone. At the same time, the monthly environmental distances also fluctuate markedly for port pairs without strong interannual temperature variation at the source port location, such as those in Australia and New Zealand, corresponding to higher risk when ballast discharges occur in Canada during the northern hemisphere’s summer and autumn (Fig. 8). This pattern supports our hypothesis that the opposite seasons in the northern and southern hemispheres may create a higher risk for vessels departing in the southern hemisphere winter (northern hemisphere summer) to arrive at Canadian ports where the overall water temperature is cooler.

Port pairs with high variability in environmental distances and high  number of ballast tank discharges were examined, with the overlap being mostly from ports in Eastern Asia to ports on the west coast of Canada (Pacific region). The sizeable temporal variation in environmental distance between the two regions is possibly a result of: 1) large inter-annual variability in sea surface temperature in the northwest pacific (Dunstan et al. 2018) (i.e., the temperate climate zone of Eastern Asia); and/or 2) salinity fluctuations at the estuaries of large rivers (Warner et al. 2005) where the ports are densely distributed. Based on the seasonal variations observed in sea surface temperature (Fig. 1) and salinity (Fig. 2), risk changes are more likely to be influenced by temperature variations in temperate climate zones, as salinity has less seasonal variability along both the west coast of Canada and Eastern Asia.

Although there have been a number of previous studies implementing environmental matching in ballast water risk assessments (Gollasch 1996; Hilliard et al. 1997; Clarke et al. 2003; Awad et al. 2004; Keller et al. 2011a), very few have analyzed the potential impacts of temporal variability in their models. Seebens et al. (2013) do demonstrate and discuss the occurrence of seasonal variability in the output of their global shipping invasion risk model, based on temporal variation in shipping intensity and temperature, though they do not quantify the difference and they continue to use annual average environmental data within their standard model. In the standardized assessment of monthly scale environmental risk conducted in this study, the factor of shipping intensity was excluded, leaving only the variability associated with the source and recipient ports environmental variables. However, in practical applications, considering shipping intensity is necessary since port pairs with moderate risk variation yet very high shipping intensity (i.e., high propagule pressure) deserve more attention than routes with significant risk variation and little shipping (i.e., low propagule pressure).

While this study examined the importance of temporal variation in environmental variables for BWRA, the results may extend more broadly to studies implementing species distribution models (SDM) to predict habitat ranges under current and future climate conditions based on environmental data associated with known occurrence/absence locations (Elith and Leathwick 2009). Both correlative and mechanistic SDM (Kearney and Porter 2009) have a strong reliance on environmental data, mainly climatic conditions. Many SDM have used environmental data at fixed spatial and temporal scales to define the distribution of species over spatial-temporal limits (Buckley et al 2010). More specifically, annual data have been used to model the range of variation in environmental variables (Tyberghein et al. 2012). In response to changes in environmental variables, some studies have proposed a combination of climate change (Williams and Jackson 2007; Austin and Van Niel 2011; Harsch and HilleRisLambers 2016) and microclimate factors (Lembrechts et al. 2019) to model species distributions. A recent study modeled the distribution of short-lived species using monthly historical data, and the proposed seasonal SDM can be better associated with habitat suitability compared to conventional SDM (Hereford et al. 2017). Similarly, the results of our work suggests the use of finer-scale data reflecting the seasonal variability of environmental variables may achieve a more accurate prediction. Since some important variables, such as temperature, experience more seasonal variation on land than in the ocean, the use of monthly or quarterly data in SDM could have even greater influence on predictions of terrestrial species invasions and range shifts.

Several future research directions could be followed to tackle remaining knowledge gaps and limitations of this study. Firstly, ballast water is known to be an important vector for introduction of NIS to freshwater ecosystems such as the Laurentian Great Lakes (Briski et al. 2011; Bailey 2015). Inland ports were excluded from this analysis due to the lack of environmental data near these ports in the World Ocean Atlas dataset; future work could include a seasonal assessment of environmental risk for ballast water discharges at inland ports if suitable data are available elsewhere. In addition, if finer scale global data are available for salinity, it would be desirable to further assess the temporal sensitivity of the environmental matching approach since salinity can fluctuate widely within a day at ports within estuaries subject to tidal influences. Moreover, the ballast tank records being fitted to models in this work span from 2019 to 2020, and are limited to discharges within Canadian waters. Although this research found similar patterns across two years, the generality of the patterns observed in this study could be examined across a wider geographic scope and time span. Nonetheless, the results of this study suggest future evaluations incorporating ballast water uptake and discharge dates (or ships’ departure and arrival dates, if the former are not available) can provide a more sensitive assessment of risk reflecting seasonal variability compared to an annual average risk model.

## Data Availability

The port dataset compiled for this study, with corresponding annual and monthly temperature and salinity data, is available via the Dryad Digital Repository at https://doi.org/10.5061/dryad.76hdr7t1k The full environmental dataset obtained from WOA 2018 can be accessed at: https://www.ncei.noaa.gov/access/world-ocean-atlas-2018/.

## Appendix 1: Port pairs with more shipping connections

See Table 4.

**Table 4:** Port pairs with large number of (more than 250) ballast tank connections in 2019–2020

Source port	Source region	Recipient port	Discharge region	Number of tanks
Boston (USA)	Northern America	Saint John (CAN)	Atlantic	2204
Portland (ME USA)	Northern America	Saint John (CAN)	Atlantic	1416
Providence	Northern America	Saint John (CAN)	Atlantic	897
Zhoushan	Eastern Asia	Vancouver (CAN)	Pacific	854
Stockton	Northern America	Vancouver (CAN)	Pacific	772
Redwood City	Northern America	Port McNeill	Pacific	660
Rizhao	Eastern Asia	Vancouver (CAN)	Pacific	615
Qingdao	Eastern Asia	Vancouver (CAN)	Pacific	608
antong	Eastern Asia	Vancouver (CAN)	Pacific	606
Los Angeles	Northern America	Vancouver (CAN)	Pacific	488
New York	Northern America	Come by Chance	Atlantic	408
Shanghai	Eastern Asia	Vancouver (CAN)	Pacific	395
Kashima	Eastern Asia	Vancouver (CAN)	Pacific	395
Chiba	Eastern Asia	Vancouver (CAN)	Pacific	393
New Haven	Northern America	Saint John (CAN)	Atlantic	393
Bayway	Northern America	Whiffen Head	Atlantic	387
Searsport	Northern America	Saint John (CAN)	Atlantic	380
Bayway	Northern America	Point Tupper	Atlantic	372
Lianyungang	Eastern Asia	Vancouver (CAN)	Pacific	370
Bayuquan	Eastern Asia	Vancouver (CAN)	Pacific	366
Lanshan	Eastern Asia	Vancouver (CAN)	Pacific	366
Baltimore, USA	Northern America	Halifax	Atlantic	313
Seattle	Northern America	Vancouver (CAN)	Pacific	306
Baltimore, USA	Northern America	Saint John (CAN)	Atlantic	304
Nagoya	Eastern Asia	Vancouver (CAN)	Pacific	302
Long Beach	Northern America	Port McNeill	Pacific	301
Providence	Northern America	Paspebiac	Atlantic	296
Long Beach	Northern America	Vancouver (CAN)	Pacific	288
Portland (OR USA)	Northern America	Vancouver (CAN)	Pacific	285
Portsmouth (NH USA)	Northern America	Saint John (CAN)	Atlantic	282
Mizushima	Eastern Asia	Vancouver (CAN)	Pacific	279
Dangjin	Eastern Asia	Roberts Bank	Pacific	275
San Francisco (USA)	Northern America	Vancouver (CAN)	Pacific	267
Caofeidian Port	Eastern Asia	Vancouver (CAN)	Pacific	259
Bucksport	Northern America	Saint John (CAN)	Atlantic	256

## Appendix 2: Distribution of the source ports

Figure 9 shows the distribution of the 602 source ports in this study. The different colors mark the 14 world regions in which the source ports are located, following the ISO-3166 standard (ISO 2020).
